# BTK inhibition is a potent approach to block IgE‐mediated histamine release in human basophils

**DOI:** 10.1111/all.13166

**Published:** 2017-04-20

**Authors:** D. Smiljkovic, K. Blatt, G. Stefanzl, Y. Dorofeeva, C. Skrabs, M. Focke‐Tejkl, W. R. Sperr, U. Jaeger, R. Valenta, P. Valent

**Affiliations:** ^1^ Department of Internal Medicine I Division of Hematology & Hemostaseology Medical University of Vienna Vienna Austria; ^2^ Ludwig Boltzmann Cluster Oncology Medical University of Vienna Vienna Austria; ^3^ Division of Immunopathology Department of Pathophysiology and Allergy Research Center for Pathophysiology, Immunology and Infectiology Medical University of Vienna Vienna Austria

**Keywords:** allergy, IgE receptor, signaling molecules, targeted drugs

## Abstract

**Background:**

Recent data suggest that Bruton's tyrosine kinase (BTK) is an emerging therapeutic target in IgE receptor (IgER)‐cross‐linked basophils.

**Methods:**

We examined the effects of four BTK inhibitors (ibrutinib, dasatinib, AVL‐292, and CNX‐774) on IgE‐dependent activation and histamine release in blood basophils obtained from allergic patients (n=11) and nonallergic donors (n=5). In addition, we examined the effects of these drugs on the growth of the human basophil cell line KU812 and the human mast cell line HMC‐1.

**Results:**

All four BTK blockers were found to inhibit anti‐IgE‐induced histamine release from basophils in nonallergic subjects and allergen‐induced histamine liberation from basophils in allergic donors. Drug effects on allergen‐induced histamine release were dose dependent, with IC_50_ values ranging between 0.001 and 0.5 μmol/L, and the following rank order of potency: ibrutinib>AVL‐292>dasatinib>CNX‐774. The basophil‐targeting effect of ibrutinib was confirmed by demonstrating that IgE‐dependent histamine release in *ex vivo* blood basophils is largely suppressed in a leukemia patient treated with ibrutinib. Dasatinib and ibrutinib were also found to counteract anti‐IgE‐induced and allergen‐induced upregulation of CD13, CD63, CD164, and CD203c on basophils, whereas AVL‐292 and CNX‐774 showed no significant effects. Whereas dasatinib and CNX‐774 were found to inhibit the growth of HMC‐1 cells and KU812 cells, no substantial effects were seen with ibrutinib or AVL‐292.

**Conclusions:**

BTK‐targeting drugs are potent inhibitors of IgE‐dependent histamine release in human basophils. The clinical value of BTK inhibition in the context of allergic diseases remains to be determined.

## 1 Introduction

Basophils (BA) and mast cells (MC) are major effector cells of anaphylactic reactions in patients suffering from IgE‐dependent allergies.^1^, ^2^, ^3^ Both cell types produce a number of biologically active mediators, including histamine, lipid mediators, and cytokines, and both cell types express high‐affinity receptors for immunoglobulin E (IgE).^1^, ^2^, ^3^, ^4^, ^5^, ^6^ Once activated by IgE receptor (IgER) cross‐linking or other stimuli, BA and MC liberate their proinflammatory substances, which leads to allergic inflammation and the clinical symptoms of anaphylaxis.^4^, ^5^, ^6^, ^7^, ^8^ The ability of BA and MC to respond to IgE‐dependent stimuli (allergens) is dependent on genetic background factors, various signal transduction molecules, and the presence of activating cytokines.^7^, ^8^, ^9^ The type and severity of an IgE‐dependent (anaphylactic) reaction depends on additional variables, including the source and configuration of the allergen, organ‐specific factors, and the numbers of BA and MC involved in the reaction.^10^, ^11^, ^12^, ^13^, ^14^ BA and MC may increase in number in various immunologic diseases, in certain types of (chronic) infectious diseases, and in distinct hematologic neoplasms. Likewise, in patients with systemic mastocytosis (SM), the numbers of MC increase substantially in various organs.^3^, ^12^, ^13^ When these patients are suffering from a concomitant IgE‐dependent allergy, anaphylactic reactions may be severe or even life‐threatening.^12^, ^13^

IgER‐dependent activation of BA and MC is accompanied by an increase in certain cell surface antigens, including CD63 and CD203c, and by activation of numerous downstream signaling pathways and molecules, including LYN, SYK, RAS, MAP kinases, PI3 kinase (PI3K), mTOR, and protein kinase C.^4^, ^5^, ^6^, ^15^, ^16^, ^17^, ^18^, ^19^, ^20^, ^21^, ^22^, ^23^, ^24^, ^25^, ^26^, ^27^ The Bruton's tyrosine kinase (BTK) has been identified as another important downstream target in IgER‐cross‐linked BA.^27^, ^28^, ^29^, ^30^, ^31^ In particular, it has been described that IgER cross‐linking in BA is followed by phosphorylation of SYK and that SYK, once activated, is capable of phosphorylating BTK.^27^, ^28^, ^29^, ^30^, ^31^ In addition, it has been described that BTK inhibition is associated with reduced mediator release in human BA.^32^

The aims of this study were to explore whether BTK can serve as a therapeutic target in BA and MC and whether the BTK blockers currently used in clinical trials are able to suppress allergen‐induced (IgER‐dependent) activation and histamine release. In addition, we examined the effects of these drugs on growth of BA and MC. The results of our study show that BTK inhibition by ibrutinib is a potent approach to suppress allergen‐induced histamine release and activation in human BA.

## 2 Material and Methods

### 2.1 Monoclonal antibodies (mAb) and other reagents

The anti‐IgE mAb E124.2.8 (Dε2), the fluorescein isothiocyanate (FITC)‐labeled mAb CLB‐gran12 (CD63), and phycoerythrin (PE)‐conjugated mAb 97A6 (CD203c) were purchased from Immunotech (Marseille, France), and human IgE from Merck Millipore (Billerica, MA, USA). A detailed description of mAb is listed in Table S1. The BTK inhibitors ibrutinib (PCI‐32765), AVL‐292, and CNX‐774 were purchased from Selleck Chemicals (Riverside, CA, USA), dasatinib from ChemieTek (Indianapolis, IN, USA), and the SYK inhibitor P505‐15 from Axon Medchem (Groningen, the Netherlands). Table S2 shows the target profiles of the kinase blockers used in this study. Stock solutions of drugs were prepared by dissolving in dimethyl sulfoxide (DMSO) (Merck, Darmstadt, Germany). The recombinant (r) allergens rDer p 2 and rPhl p 5 were obtained from Biomay (Vienna, Austria). Histamine release buffer (HRB) and histamine radioimmunoassay (RIA) kit were purchased from Immunotech, and RPMI 1640 medium, Iscove's modified Dulbecco's medium (IMDM), and fetal calf serum (FCS) from Thermo Fisher Scientific (Waltham, MA, USA).

### 2.2 Isolation of blood BA

Peripheral blood was obtained from 5 healthy individuals and 11 patients allergic to Der p 2 and/or Phl p 5. Patients were diagnosed according to standard diagnostic procedures and their molecular IgE reactivity profiles were determined by ISAC (immuno‐solid‐phase allergen chip) technology.^33^ The patients’ characteristics are shown in Table S3. Informed consent was obtained in each case. The study was approved by the ethics committee of the Medical University of Vienna (EK1641/2014) and conducted in accordance with the Declaration of Helsinki. Peripheral blood was collected in heparin‐containing tubes. BA were enriched by dextran sedimentation (histamine release experiments) or were recovered together with mononuclear cells (MNC) after centrifugation over Ficoll (immunostaining experiments) as described.^34^ The percentage of BA ranged from 0.1% to 1.5% in dextran preparations, and from 0.3% to 2% in MNC. Cell viability was >90% as assessed by trypan blue exclusion test.

### 2.3 Cell lines

The human MC line HMC‐1 was kindly provided by Dr. J.H. Butterfield (Mayo Clinic, Rochester, MN, USA). Two subclones were used, HMC‐1.1 expressing KIT V560G and HMC‐1.2 expressing KIT V560G and KIT D816V.^35^, ^36^ HMC‐1 cells were grown in IMDM with 10% FCS, alpha‐thioglycerol (Sigma), and antibiotics. The human BA cell line KU812 was kindly provided by Dr. K. Kishi (Kumamoto University, Kumamoto, Japan) and cultured in RPMI 1640 medium with 10% FCS.^37^

### 2.4 Histamine release assay

The histamine release assay was performed on dextran‐enriched BA (healthy donors, n=5; allergic donors, n=11) essentially as described.^18^, ^38^, ^39^ Dextran‐enriched BA were incubated in the presence or absence of various concentrations of targeted drugs (dasatinib, ibrutinib, AVL‐292, CNX‐774, P505‐15) (0.001‐1 μmol/L) for 30 minutes at 37°C, and thereafter incubated with anti‐IgE antibody E124.2.8 (1 μg/mL) in HRB at 37°C for another 30 minutes. Drug‐exposed BA from allergic patients were incubated with recombinant Der p 2 and Phl p 5 (each 1 μg/mL) or control buffer (HRB) for 30 minutes. In select experiments, BA were preincubated with ibrutinib (1.0 μmol/L) and then exposed to Der p 2 (0.001‐10 μg/mL). After incubation, cells were centrifuged at 4°C, and the cell‐free supernatants and total suspensions recovered and analyzed for histamine content by RIA. Histamine release was calculated and expressed as percentage of total histamine. All experiments were performed in triplicates. In a separate set of experiments, anti‐IgE‐induced histamine release from BA was examined in a patient with chronic lymphocytic leukemia (CLL) treated with ibrutinib (280 mg/day per os). In this experiment, BA were obtained before treatment with ibrutinib and 14 days after the start of therapy. *ex vivo* obtained BA were incubated in HRB in the absence or presence of anti‐IgE antibody E‐124.2.8 (0.001‐10 μg/mL) at 37°C for 30 minutes. Then, histamine release was measured as described above.

### 2.5 Antibody staining experiments and flow cytometry

Whole‐blood cells were incubated with various tyrosine kinase inhibitors (TKI: dasatinib, ibrutinib, AVL‐292, CNX‐774, and P505‐15) (0.001‐10 μmol/L) at 37°C for 30 minutes. Then, cells were washed and incubated with anti‐IgE mAb E124.2.8 (1 μg/mL) or allergens (1 μg/mL) together with fluorochrome‐labeled mAb against CD13, CD63, CD164, or CD203c for 15 minutes. Thereafter, cells were subjected to erythrocyte lysis and analyzed by multicolor flow cytometry on a FACSCalibur as described.^18^, ^38^, ^40^ BA were identified as CD203c‐positive cells. The anti‐IgE‐induced or allergen‐induced upregulation of CD13, CD63, CD164, and CD203c on BA was calculated from mean fluorescence intensities (MFI) obtained with stimulated (MFI_stim_) and unstimulated (MFI_control_) cells, and expressed as stimulation index, SI (MFI_stim_:MFI_control_).^18^, ^38^, ^40^ To explore drug effects on baseline expression of CD63 and/or CD203c in HMC‐1 and KU812, cells were incubated with dasatinib, ibrutinib, AVL‐292, CNX‐774, P505‐15 (each 0.01‐10 μmol/L), or control medium at 37°C for 24 hours. Then, expression of CD63 and CD203c was analyzed on a FACSCalibur. All staining reactions were controlled by isotype‐matched antibodies. For staining of cytoplasmic molecules, HMC‐1 and KU812 cells were incubated in dasatinib, ibrutinib, AVL‐292, CNX‐774, P505‐15 (0.1‐10 μmol/L), or control medium at 37°C for 4 hours. Then, cells were permeabilized by methanol (−20°C, 15 minutes) and incubated with mAb against pBTK, pSYK, pAKT, pS6, pSTAT5, or active caspase 3 for 30 minutes.^40^ Thereafter, cells were washed and analyzed on a FACSCalibur. In a separate set of experiments, BA‐containing MNC were incubated with TKI (dasatinib, ibrutinib, AVL‐292, CNX‐774, P505‐15; 0.1‐10 μmol/L) at 37°C for 15 minutes. Then, cells were washed and incubated with anti‐IgE for another 15 minutes. For the detection of intracellular pBTK and pSYK, intact cells were first incubated with an APC‐labeled mAb against CD203c or a PE‐labeled mAb against CD203c for 15 minutes, washed, and then permeabilized with methanol.^40^ Thereafter, cells were stained with an Alexa Fluor647‐conjugated antibody against pBTK or a PE‐labeled mAb against pSYK (30 minutes). Expression of intracellular targets in CD203c^+^ BA was quantified by multicolor flow cytometry on a FACSCalibur as reported.^40^ Apoptosis was measured in drug‐exposed cells by combined AnnexinV/propidium iodide (PI) staining following a published protocol.^36^, ^40^ For cell cycle studies, drug‐exposed cells were resuspended in 500 μL permeabilization buffer. Then, 40 μL PI was added and cell cycle distribution was analyzed on a FACSCalibur as described previously.^41^

### 2.6 Measurement of ^3^H‐thymidine uptake

HMC‐1 cells and KU812 cells were incubated in control medium or in various concentrations of ibrutinib, AVL‐292, CNX‐774, or P505‐15 (range: 0.001‐10 μmol/L) or dasatinib (0.000001‐10 μmol/L) at 37°C for 48 hours. Thereafter, 0.5 μCi ^3^H‐thymidine was added (37°C, 16 hours). Cells were then harvested on filter membranes in a Filtermate 196 harvester (Perkin Elmer, Waltham, MA, USA). Filters were air‐dried, and the bound radioactivity was counted in a β‐counter (MicroBeta^2^ 2450 Microplate Counter; Perkin Elmer). All experiments were performed in triplicates.

### 2.7 Statistical analysis

To determine the level of significance in drug incubation experiments, histamine release experiments and surface staining experiments in BA and human cell lines, the paired Student's *t* test was applied. In case of multiple comparisons, the Bonferroni correction was performed. A *P* value of <0.05 was considered to indicate statistical significance.

## 3 Results

### 3.1 Effects of targeted drugs on IgER downstream signaling molecules

**Figure 1:**
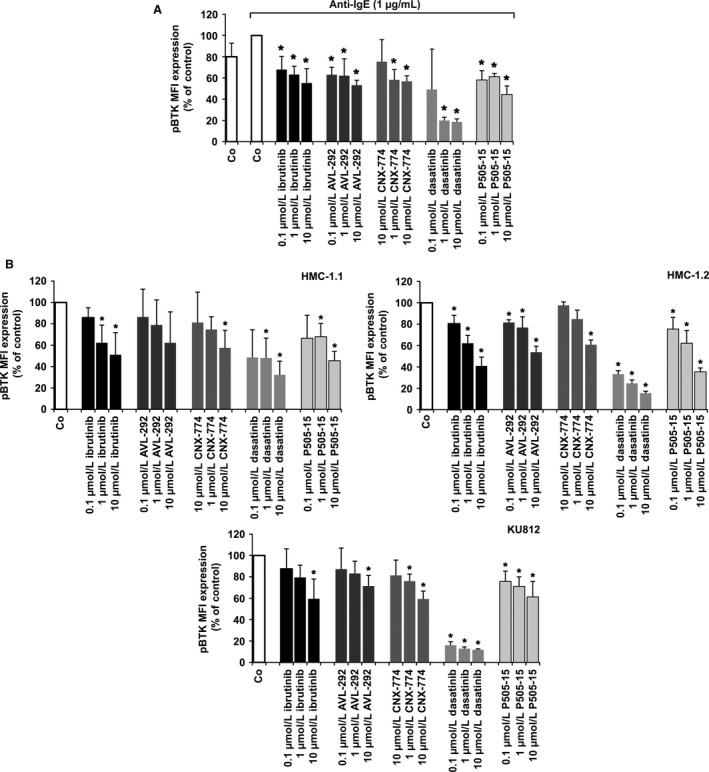
Effects of ibrutinib on expression of pBTK in primary activated human basophils (BA), HMC‐1 cells, and KU812 cells. (A) BA‐containing mononuclear cells (MNC) were incubated in control medium (Co) or in medium containing dasatinib, ibrutinib, AVL‐292, CNX‐774, or P505‐15 (each 0.1‐10 μmol/L) at 37°C for 15 minutes. Then, cells were incubated with anti‐IgE at 37°C for 15 minutes and incubated with a PE‐labeled mAb against CD203c for 15 minutes. Then, cells were permeabilized and stained with an antibody against phosphorylated (p) BTK (pBTK) (phosphorylation site: Y223) as described in the text. Expression of intracellular targets was quantified by multicolor flow cytometry on a FACSCalibur. BA were identified as CD203c‐positive cells. Results show the mean fluorescence intensity (MFI) of pBTK expression (% of control) and represent mean±SD from three independent experiments. Asterisk (*) *P*<0.05 by Student's *t* test with Bonferroni correction. (B) HMC‐1.1 cells (upper left panel), HMC‐1.2 cells (upper right panel), and KU812 cells (lower panel) were incubated with control medium (Co) or medium containing ibrutinib, AVL‐292, CNX‐774, dasatinib, or P505‐15 (0.1‐10 μmol/L) at 37°C for 4 hours. Thereafter, cells were permeabilized and stained with an antibody against pBTK (Y223). Expression of phosphorylated (p) signaling molecules in HMC‐1 and KU812 cells was determined by flow cytometry. Results show MFI values expressed as percentage of control and represent the mean±SD from three independent experiments. Asterisk (*): *P*<0.05 by Student's *t* test with Bonferroni correction

To study drug effects on BTK activation and to explore the specificity of these effects, we examined the phosphorylation status of various IgER downstream signaling molecules in drug‐exposed and IgER‐cross‐linked BA as well as in untreated or drug‐exposed cell lines (HMC‐1.1, HMC‐1.2, KU812). We found that dasatinib, ibrutinib, AVL‐292, and CNX‐774 counteract anti‐IgE‐induced expression of pBTK in BA (Figure 1A). In addition, the SYK inhibitor P505‐15 was found to block expression of pBTK in BA (Figure 1A). These drugs were also found to block pBTK expression in unstimulated HMC‐1.1, HMC‐1.2, and KU812 cells (Figure 1B). In control experiments, P505‐15 also decreased expression of pSYK in IgER‐cross‐linked BA (not shown). We also found that ibrutinib as well as the other BTK blockers applied suppress expression of pSYK, pAKT, pS6, and pSTAT5 in HMC‐1 and KU812 cells (Fig. S1). Of all drugs applied, dasatinib was found to exert most potent effects on expression of pBTK and BTK downstream kinase targets, thereby confirming the broad target interaction profile of this drug.^38^

### 3.2 Ibrutinib inhibits IgE‐dependent histamine release in BA

**Figure 2:**
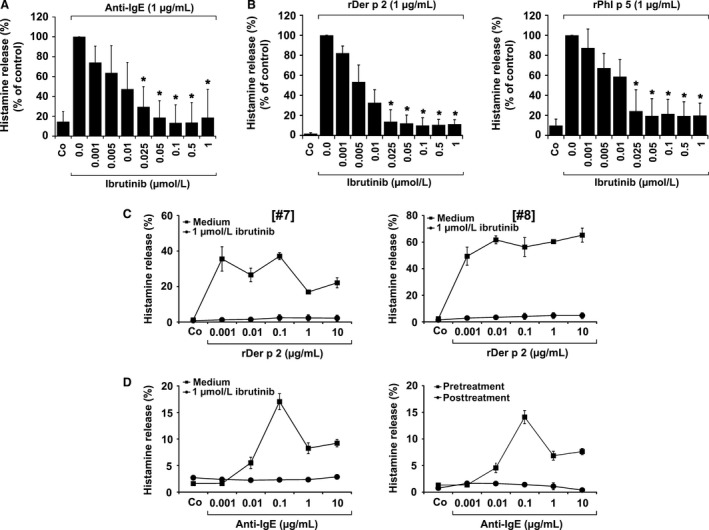
Effects of ibrutinib on IgE‐mediated histamine release in human basophils. Basophils (BA) obtained from three nonallergic donors (A) or patients allergic to Der p 2 (n=3) or Phl p 5 (n=3) (B) were preincubated in control medium (Co) or various concentrations of ibrutinib (0.001‐1 μmol/L) at 37°C for 30 minutes. Then, cells were exposed to anti‐IgE (1 μg/mL; nonallergic donors) or recombinant allergens (1 μg/mL of rDer p 2 or rPhl p 5; allergic patients) at 37°C for 30 minutes. After centrifugation, histamine concentrations were determined in supernatants and cell lysates. Histamine release is expressed as percentage of total histamine. Results show the percentage of control and represent mean±SD of three independent experiments. Asterisk (*): *P*<0.05 by Student's *t* test. (C) BA from two patients allergic to Der p 2 (left and right panel) were incubated in control medium (Co) or 1 μmol/L ibrutinib at 37°C for 30 minutes. Then, cells were incubated in histamine release buffer (HRB) in the absence or presence of rDer p 2 (0.001‐10 μg/mL) at 37°C for 30 minutes. After incubation, cells were centrifuged at 4°C and cell‐free supernatants and cell suspensions analyzed for histamine content. Histamine release is expressed as percentage of total histamine. Results represent the mean±SD of triplicates. (D) Left panel: BA from a patient with chronic lymphocytic leukemia (CLL) were incubated in control medium (Co) or 1 μmol/L ibrutinib at 37°C for 30 minutes. Thereafter, cells were incubated in HRB in the absence or presence of anti‐IgE (0.001‐10 μg/mL) at 37°C for 30 minutes. After incubation, cells were centrifuged at 4°C and cell‐free supernatants and cell suspensions analyzed for histamine content. Histamine release is expressed as percentage of total histamine. Results represent the mean±SD of triplicates. Right panel: In the same patient with CLL, BA were obtained before therapy with ibrutinib (pretreatment; ■‐■) and 14 days after treatment with 280 mg/day ibrutinib (post‐treatment; ●‐●). Cells were incubated in HRB in the absence or presence of anti‐IgE antibody E‐124.2.8 (0.001‐10 μg/mL) for 30 minutes. Then, histamine release was measured as described above. Histamine release is expressed as percentage of total histamine. Results represent the mean±SD of triplicates.

The BTK blocker ibrutinib is already used in clinical practice and exhibits a favorable toxicity profile. In this study, ibrutinib was found to inhibit IgE‐dependent histamine release in BA obtained from healthy donors (Figure 2A, Table S4) as well as allergen‐induced histamine secretion in BA of allergic patients (Figure 2B, Table S3). The effects of ibrutinib were dose dependent, with IC_50_ values ranging between 0.003 and 0.03 μmol/L (0.01±0.01 μmol/L) in healthy donors (Table S4) and between 0.003 and 0.023 μmol/L (0.01±0.01 μmol/L) in allergic patients (Table S3). The inhibitory effects of ibrutinib (1 μmol/L) were seen at all allergen concentrations applied (Figure 2C). We also examined *ex vivo* BA obtained from a patient before and during treatment with ibrutinib, and found that ibrutinib therapy is followed by downregulation of IgE‐dependent histamine release in BA (Figure 2D). These data show that ibrutinib is a potent inhibitor of IgE‐dependent secretion of histamine in BA.

### 3.3 Effects of other BTK‐targeting drugs on IgE‐dependent histamine release

**Figure 3:**
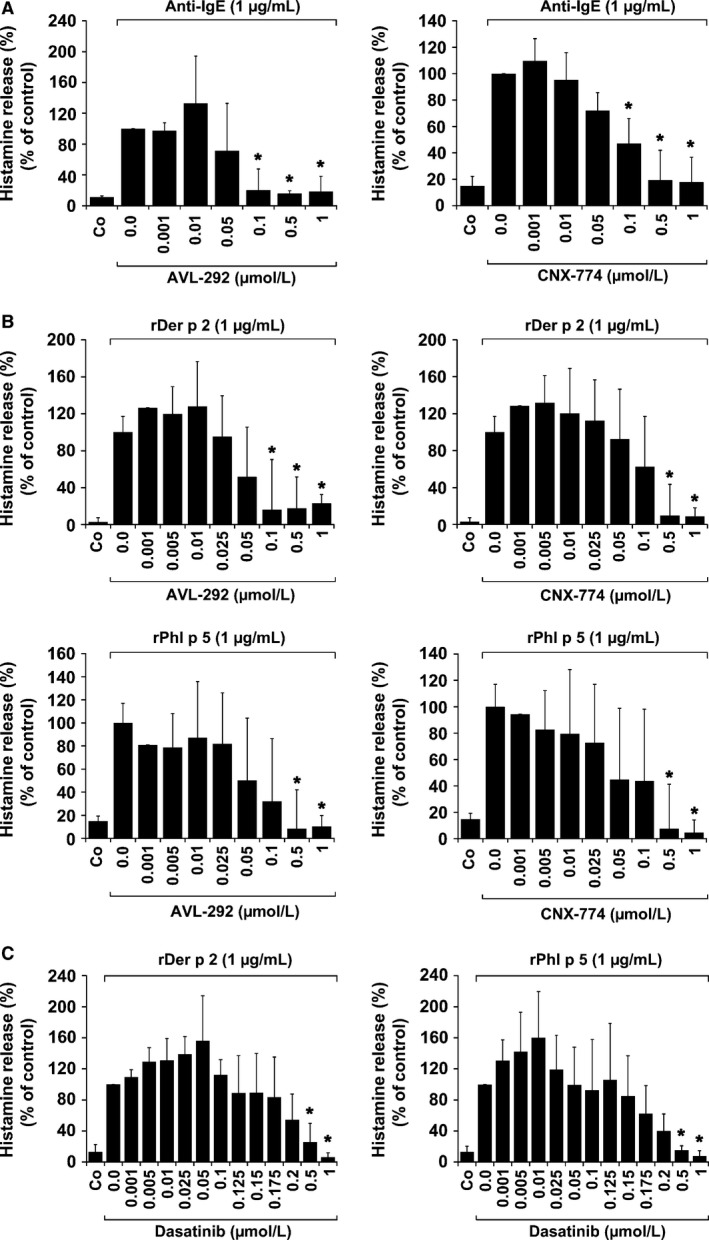
Effects of AVL‐292, CNX‐774 and dasatinib on IgE‐mediated histamine release in human basophils. Basophils (BA) obtained from three nonallergic donors (A), three patients allergic to Der p 2, and three patients allergic to Phl p 5 (B and C) were preincubated in control medium (Co) or medium containing various concentrations of AVL‐292, CNX‐774, or dasatinib (0.001‐1 μmol/L) at 37°C for 30 minutes. Then, cells were exposed to anti‐IgE antibody E‐124.2.8 (1 μg/mL; nonallergic donors) or recombinant allergens (1 μg/mL of rDer p 2 or rPhl p 5 in allergic patients) at 37°C for 30 minutes. After centrifugation, histamine concentrations were determined in cell‐free supernatants and cell lysates. Histamine release is expressed as percentage of total histamine. Results show the percentage of control and represent mean±SD from three independent experiments (three donors). Asterisk (*): *P*<0.05 by Student's *t* test with Bonferroni correction

In a next step, we confirmed the value of BTK as a potential drug target by applying other BTK blockers. In these experiments, AVL‐292 and CNX‐774 were found to counteract IgE‐mediated and allergen‐induced histamine release in IgER‐cross‐linked BA in a dose‐dependent manner (Figure 3A,B). A summary of IC_50_ values obtained with BTK inhibitors is shown in Table S4 (nonallergic individuals) and Table S5 (Der p 2‐ and Phl p 5‐allergic donors). Dasatinib was also applied as positive control and found to block allergen‐induced histamine release at higher concentrations (Figure 3C), confirming our previous results.^38^ However, at lower concentrations, dasatinib even promotes histamine release.^38^ Of all drugs tested, ibrutinib was found to be the most potent inhibitor of allergen‐induced histamine release in human BA (Table S5).

### 3.4 Effects of ibrutinib on IgE‐dependent upregulation of CD13, CD63, CD164, and CD203c on BA

**Figure 4:**
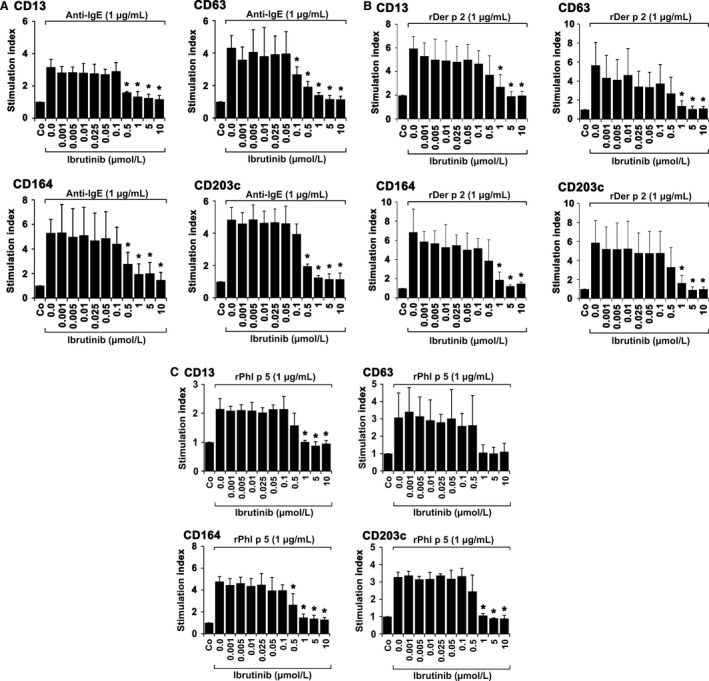
Effects of ibrutinib on expression of activation‐linked cell surface antigens on human blood basophils. Basophils (BA) obtained from three nonallergic donors (A), three patients allergic to Der p 2 (B), and three allergic to Phl p 5 (C) were preincubated in control medium (Co) or in medium containing various concentrations of ibrutinib (0.001‐10 μmol/L) at 37°C for 30 minutes. Then, cells were exposed to anti‐IgE antibody E‐124.2.8 (1 μg/mL; healthy donors) or recombinant allergens (1 μg/mL of rDer p 2 or rPhl p 5; allergic patients) for another 15 minutes (37°C). Thereafter, cells were stained with monoclonal antibodies against CD13, CD63, CD164, or CD203c and analyzed by multicolor flow cytometry as described in the text. BA were defined as CD203c^+^ cells. Anti‐IgE‐ or allergen‐induced upregulation of CD antigens was determined from mean fluorescence intensities (MFI) obtained with stimulated (MFIstim) and unstimulated (MFIcontrol) cells and expressed as stimulation index (SI=MFIstim: MFIcontrol). Results show SI values and represent the mean±SD from three donors in each experiment. Asterisk (*): *P*<0.05 by Student's *t* test with Bonferroni correction

IgER‐dependent activation of BA is accompanied by an increased expression of activation‐linked cell surface antigens.^15^, ^16^, ^17^, ^18^, ^19^, ^20^, ^21^ In this study, we found that ibrutinib dose dependently inhibits anti‐IgE‐induced upregulation of CD13, CD63, CD164, and CD203c on normal human BA, with IC_50_ values ranging between 0.1 and 0.5 μmol/L (Figure 4A). In addition, ibrutinib was found to inhibit allergen‐induced upregulation of CD13, CD63, CD164, and CD203c on BA obtained from allergic patients, with similar IC_50_ values (0.1‐0.5 μmol/L) (Figure 4B,C). Dasatinib was also found to inhibit IgE‐mediated upregulation of CD63 and CD203c on BA, confirming previous data.^38^ Unexpectedly, however, AVL‐292 and CNX‐774 did not modulate IgE‐dependent upregulation of activation‐linked cell surface antigens on BA (not shown).

### 3.5 Effects of BTK‐targeting drugs on proliferation in basophil and mast cell lines

**Figure 5:**
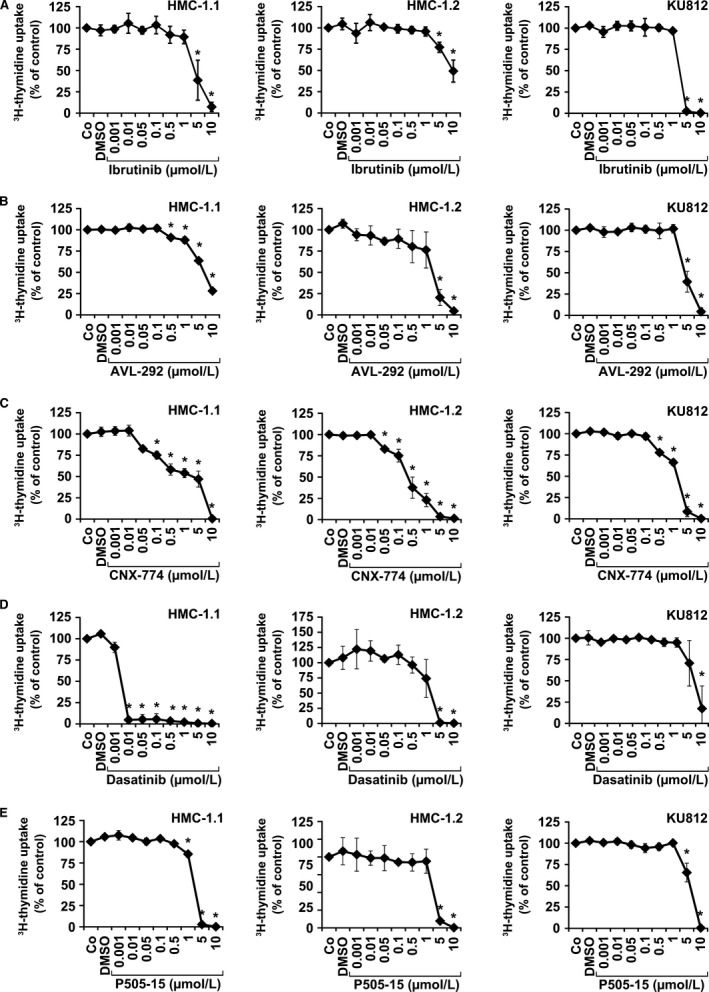
Effects of ibrutinib, AVL‐292, and CNX‐774 on proliferation in HMC‐1 cells and KU812 cells. HMC‐1.1 cells, HMC‐1.2 cells, and KU812 cells were cultured in control medium (Co), control medium containing DMSO 1:1000 (DMSO), or with increasing concentrations of ibrutinib (0.001‐10 μmol/L) (A), AVL‐292 (0.001‐10 μmol/L) (B), CNX‐774 (0.001‐10 μmol/L) (C), dasatinib (0.000001‐10 μmol/L) (D), or P505‐15 (0.001‐10 μmol/L) (E) at 37°C for 48 hours. Thereafter, ³H‐thymidine uptake was measured. Results show the percentage of ³H‐thymidine uptake compared to control (Co) and represent the mean±SD of three independent experiments in each cell line. Asterisk (*): *P*<0.05 by Student's *t* test after Bonferroni correction

As proliferation of normal and neoplastic MC is triggered by KIT activation and BTK may also serve as KIT downstream target, we examined the effects of ibrutinib and other BTK blockers on proliferation of human BA and MC lines. As determined by ^3^H‐thymidine uptake, ibrutinib, AVL‐292, and P505‐15 showed no significant effects on proliferation of HMC‐1.1, HMC‐1.2, and KU812 cells unless high concentrations (≥1 μmol/L) were applied (Figure 5A,B,E and Table 1). However, unexpectedly, CNX‐774 was found to counteract proliferation of HMC‐1.1 and HMC‐1.2 cells at relatively low concentrations (IC_50_ in HMC‐1.2: 0.1‐0.5 μmol/L) (Figure 5C and Table 1). Dasatinib, a TKI known to block wild‐type KIT and KIT D816V, was found to inhibit growth of HMC‐1 and KU812 cells in a dose‐dependent manner. IC_50_ values obtained with KU812 (0.0007 μmol/L) and HMC‐1.1 cells were lower compared to that obtained with HMC‐1.2 cells (1.45 μmol/L) (Figure 5D and Table 1).

**Table 1:** Effects of various targeted drugs on proliferation of HMC‐1 cells and KU812 cells

Cell lines	Inhibitory effects of drugs on proliferation (IC_50_, μmol/L)				
	Ibrutinib	AVL‐292	CNX‐774	Dasatinib	P505‐15
HMC‐1.1	4.45	6.91	2.82	0.005	2.70
HMC‐1.2	9.04	2.73	0.38	1.45	3.03
KU812	2.98	4.42	1‐5	0.0007	6.10

HMC‐1 cells and KU812 cells were incubated in increasing concentrations of drugs (range for tested compounds: 0.001‐10 μmol/L; range for dasatinib on KU812 cells: 0.001‐10 nmol/L) at 37°C for 48 hours. Proliferation was measured by analyzing ^3^H‐thymidine uptake. Results are expressed as inhibitory concentration producing 50% inhibition (IC_50_).

### 3.6 Effects of CNX‐774 on cell cycle distribution and apoptosis in human BA and MC lines

We also examined CNX‐774‐exposed HMC‐1 and KU812 cells for alterations in cell cycle distribution and signs of apoptosis. In these experiments, CNX‐774 was found to exhibit no effect on cell cycle progression in the three cell lines tested (Fig. S2A). CNX‐774 induced an increase in apoptotic cells as determined by staining for AnnexinV/PI and active caspase‐3 in the three cell lines examined (Fig. S2B). However, this effect of CNX‐774 was only seen at relatively high concentrations. Dasatinib also induced apoptosis in all three cell lines examined (not shown), confirming previous studies.^41^

## 4 Discussion

Allergen‐induced and IgE‐dependent activation of BA and MC and the consecutive release of vascular and proinflammatory mediators from these cells are key events in allergic reactions.^4^, ^5^, ^6^, ^7^, ^8^ However, although several relevant signaling molecules and pathways downstream of the IgER have been identified and many different drugs are available, little is known about the effects of these agents on BA. We describe that the BTK blocker ibrutinib is a most potent inhibitor of allergen‐induced activation and histamine release in human BA. In addition, we show that other irreversible BTK blockers, including dasatinib,^42^ also inhibit allergen‐induced (IgE‐dependent) histamine release. The effects of these BTK inhibitors were dose dose‐dependent and occurred at pharmacological concentrations which may have clinical implications and may pave the way to the development of new antiallergic treatment concepts in patients with IgE‐dependent allergies.

Recent data suggest that ibrutinib blocks IgE‐dependent activation and histamine release in normal blood BA.^32^ In the present study, we were able to confirm this effect of ibrutinib. In addition, our data show that ibrutinib is also a potent inhibitor of allergen‐induced activation and histamine release in BA obtained from allergic donors. The IC_50_ values in normal BA (nonallergic donors) and allergen‐exposed BA obtained from patients allergic to Der p 2 and Phl p 5 were comparable and were found to be within a pharmacologically meaningful range, suggesting that the drug may indeed be able to block histamine secretion in patients with IgE‐dependent allergies. This hypothesis was confirmed by studies on *ex vivo* BA obtained from a CLL patient receiving ibrutinib. In this experiment, IgE‐dependent histamine release in *ex vivo* obtained BA was almost completely suppressed during therapy compared to pretreatment results.

A number of previous and more recent data suggest that BTK is an essential IgER downstream regulator of activation and mediator secretion in BA and MC.^32^ It has also been described that BTK is downstream of SYK and upstream of several other major kinase targets relevant to IgE‐dependent activation of BA and MC.^43^ However, the exact routes in the signaling cascades downstream of BTK in BA and MC are currently unknown. In the present study, we confirmed that ibrutinib downregulates phosphorylation and thus activation of BTK in IgER‐cross‐linked BA. However, we also found that ibrutinib blocks the activity (phosphorylation) of several other downstream kinases, including AKT, S6, and STAT5. As ibrutinib is known to recognize also several other kinase targets, such as LYN, FYN, SRC, and ITK, it may well be that some of the inhibitory effects of ibrutinib were not exerted through BTK but via suppression of other target kinases.^43^ An alternative explanation would be that some of these targets are activated by BTK in BA and that the ibrutinib‐induced effects on these additional targets were (in part) mediated through BTK inhibition. It is generally appreciated that ibrutinib is not recognizing SYK. Correspondingly, ibrutinib did not block phosphorylation of SYK in BA in our study. By contrast, the SYK inhibitor P505‐15 was found to block phosphorylation of SYK in BA and to suppress histamine release, confirming our previous results.^44^ As BTK is downstream of SYK, we also examined the effects of P505‐15 on BTK activation. Indeed, as expected, P505‐15 was found to suppress BTK activation in BA. Whether P505‐15 blocks histamine secretion through BTK disruption or also in a BTK‐independent manner remains unknown. We also applied other BTK inhibitors in order to confirm the role of BTK in IgE‐dependent activation and histamine secretion. All BTK blockers examined, including dasatinib, were found to suppress IgE‐dependent histamine secretion in BA. With regard to dasatinib, these results confirm our previous data.^38^, ^42^ However, of all drugs tested, the most potent blocker of allergen‐induced histamine secretion in BA appears to be ibrutinib.

Recent data suggest that ibrutinib inhibits IgE‐dependent upregulation of CD63 and CD203c on normal BA.^32^ In the current study, we confirmed this drug effect. In addition, we were able to show that ibrutinib blocks allergen‐induced upregulation of CD63 and CD203c in BA obtained from patients allergic to Der p 2 and/or Phl p5, with comparable IC_50_ values. In addition, we found that ibrutinib inhibits IgE‐dependent upregulation of CD13 and CD164 on BA. Unexpectedly, however, the other BTK inhibitors tested did not counteract upregulation of CD13, CD63, CD164, or CD203c on BA. From these data, one could speculate that the effects of ibrutinib on IgE‐dependent upregulation of these activation antigens were mediated by other (additional) drug targets in BA. Alternatively, the inhibitory effects of the other drugs on BTK activation were too weak to result in downregulation of these CD molecules.

The severity of an allergic reaction may not only depend on the type of allergen, IgE levels, cytokine exposure, and the microenvironment but also on the total number of effector cells involved in the reaction.^12^, ^13^, ^14^ Conditions with high numbers of BA and MC include chronic inflammation, certain neoplastic states, and specific neoplasms, including basophilic leukemias and mastocytosis. In these patients, BA and MC progenitors have a significant proliferative potential.^3^, ^12^, ^13^ We were therefore interested to learn whether ibrutinib and/or the other drugs tested would exert effects on growth of human BA and MC. To address this point, human BA and MC lines were employed. We found that dasatinib and CNX‐774 inhibit growth of HMC‐1.1 cells and KU812 cells at relatively low drug concentrations. In HMC‐1.2 cells, both drugs were also effective, but the concentrations required to block cell growth were rather high. This phenomenon may be explained by the fact that HMC‐1.2 cells display KIT D816V and that KIT D816V downstream pathways lead to resistance. In the case of dasatinib, KIT D816V itself may be responsible for the weaker effect of the drug in HMC‐1.2 cells compared to HMC‐1.1 cells lacking KIT D816V. The other drugs tested did not exert substantial effects on growth and proliferation of KU812 cells or HMC‐1 cells, suggesting that BTK is no relevant mediator of proliferation of BA and MC progenitors.

In summary, our data show that ibrutinib is a most potent inhibitor of allergen‐induced (IgE‐dependent) activation and histamine release in human BA and MC. The effects of the drug were seen in normal BA as well as in allergen‐exposed BA obtained from patients allergic to Der p 2 or Phl p 5, with meaningful IC_50_ values. Moreover, we found that other BTK blockers also counteract mediator secretion in BA. These effects may have clinical implications and may provide a basis for the development of new treatment concepts in IgE‐dependent allergies.

## Author Contributions

D.S. performed proliferation and apoptosis assays, flow cytometry experiments, and cell activation experiments, and wrote parts of the manuscript; K.B. performed flow cytometry experiments and wrote parts of the manuscript; G.S. performed cell activation experiments and histamine measurements; Y.D., M.F.‐T., W.R.S., and R.V. contributed patient material and vital reagents; C.S., W.R.S., and U.J. contributed patient material and parts of the study design; and P.V. contributed the study conception and design, patient material, and wrote parts of the manuscript.

## Conflict of Interest

The authors declare that they have no conflict of interest in the current study. Conflict of interest unrelated to the present study: U.J. received honoraria from Janssen‐Cilag. R.V. received research grants from Biomay Ag, Vienna, Austria; Thermo Fisher, Uppsala, Sweden; and Fresenius Medical Care, Bad Homburg, Germany; and serves as a consultant for these companies. W.R.S. received a research grant from Lipomed, Arlesheim, Switzerland. P.V. received research grants from Novartis, Deciphera, and Blueprint, and honoraria from Novartis, Ariad, Pfizer, BMS, Deciphera, and Celgene.
