# Supplementary figures and images for: BTK inhibition is a potent approach to block IgE‐mediated histamine release in human basophils
Source: Allergy. 2017 Apr 20;72(11):1666–76. doi: 10.1111/all.13166 (PMC5655929; doi:10.1111/all.13166)

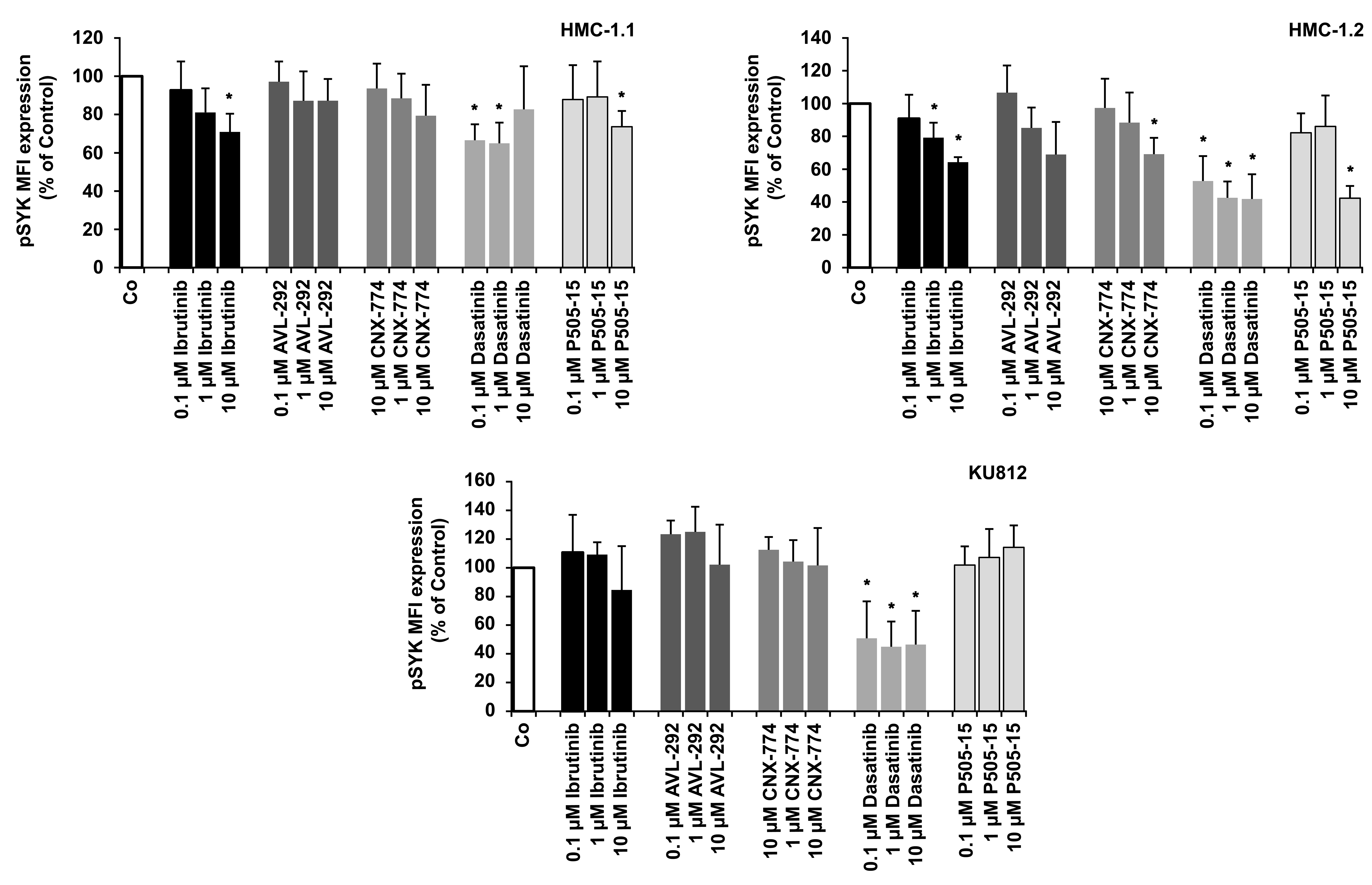

Supplement: Supplementary file 1 [file ALL-72-1666-s001.zip › all13166-sup-0002-FigS1A.tif]

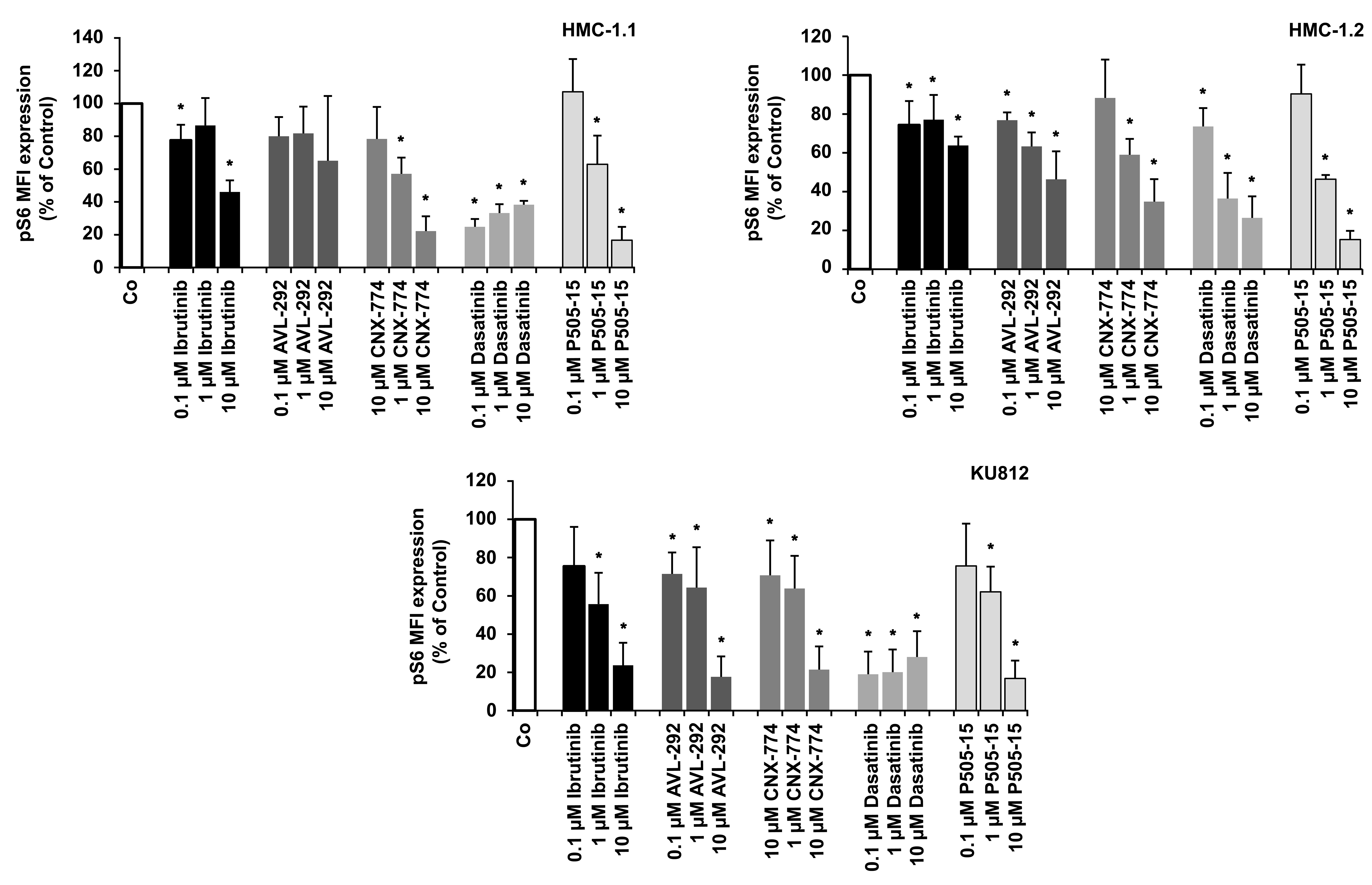

Supplement: Supplementary file 1 [file ALL-72-1666-s001.zip › all13166-sup-0003-FigS1B.tif]

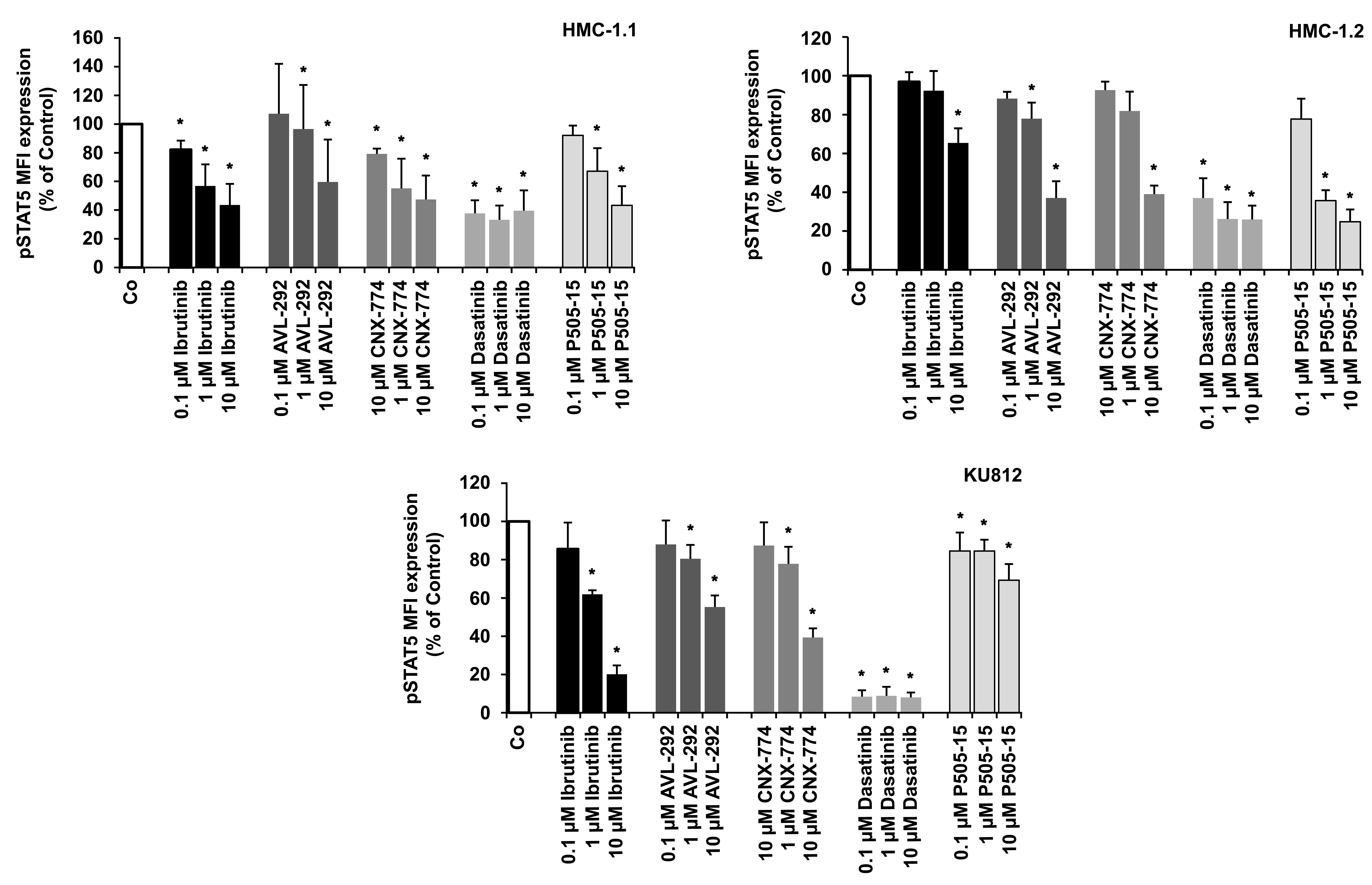

Supplement: Supplementary file 1 [file ALL-72-1666-s001.zip › all13166-sup-0004-FigS1C.tif]

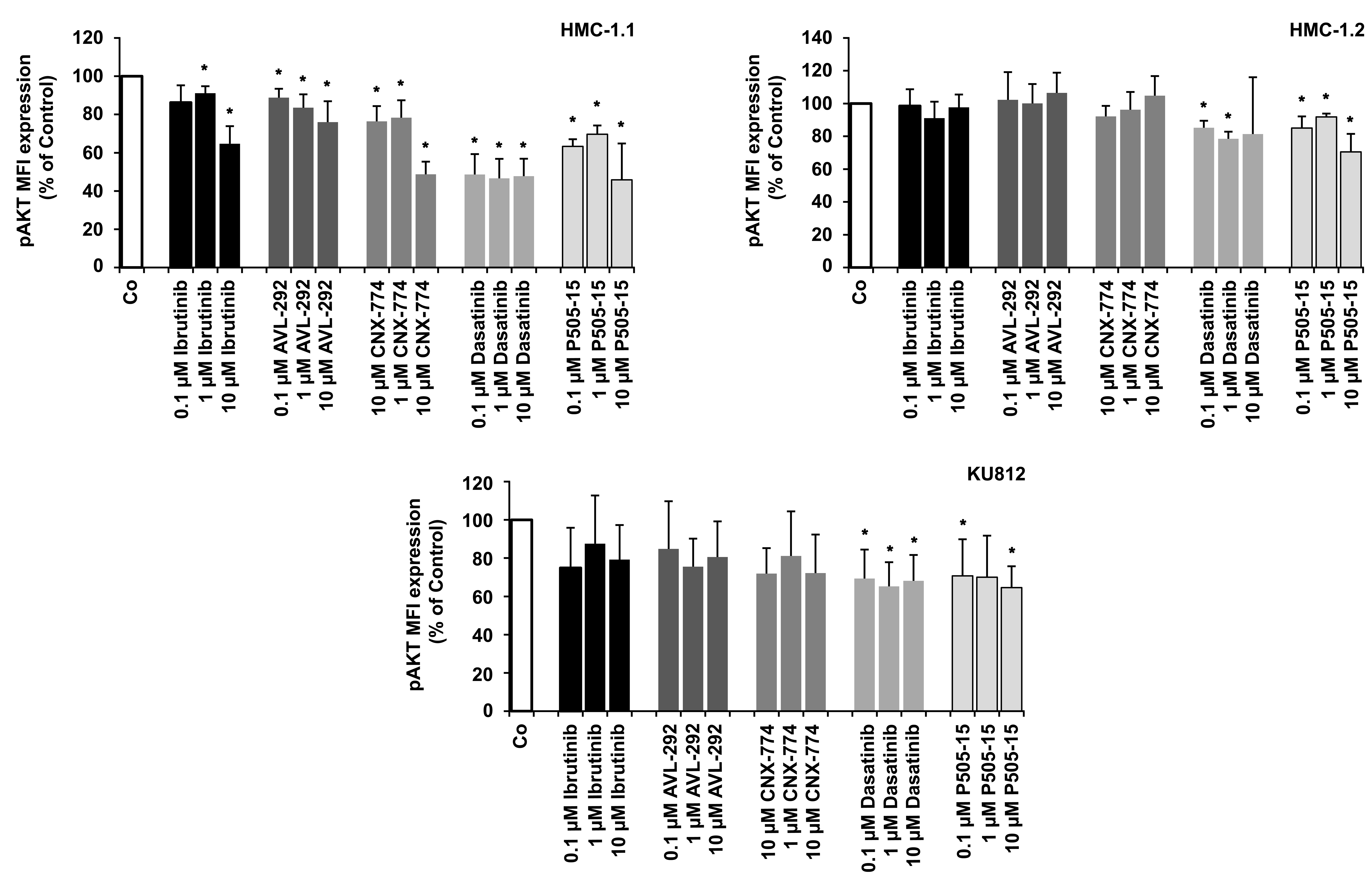

Supplement: Supplementary file 1 [file ALL-72-1666-s001.zip › all13166-sup-0005-FigS1D.tif]

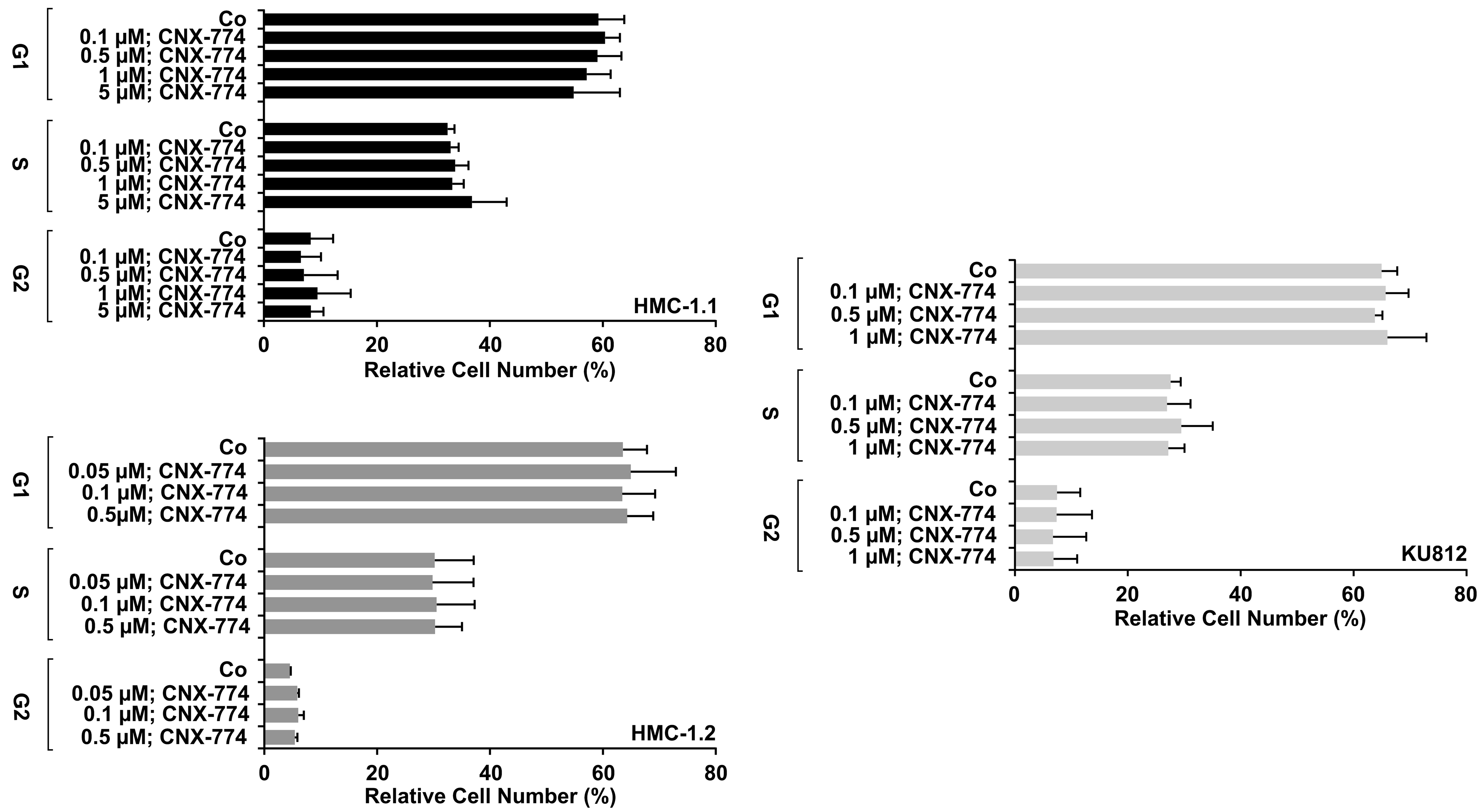

Supplement: Supplementary file 1 [file ALL-72-1666-s001.zip › all13166-sup-0006-FigS2A.tif]

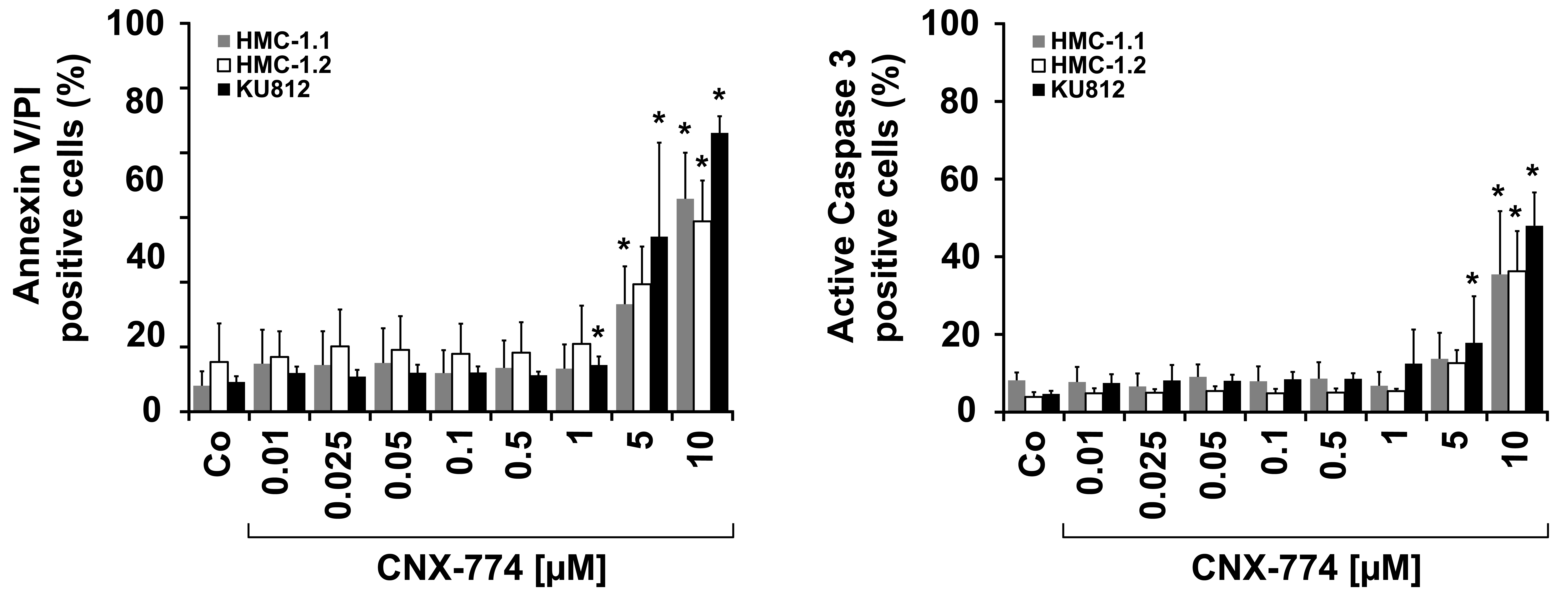

Supplement: Supplementary file 1 [file ALL-72-1666-s001.zip › all13166-sup-0007-FigS2B.tif]
