# Supplementary material for: BTK inhibition is a potent approach to block IgE‐mediated histamine release in human basophils
Source: Allergy. 2017 Apr 20;72(11):1666–76. doi: 10.1111/all.13166 (PMC5655929; doi:10.1111/all.13166)
Supplement: Supplementary file 1 [file ALL-72-1666-s001.zip › all13166-sup-0001-Suppinfo.doc]

**Supplementary Material to Manuscript: Smiljkovic et al:**

**BTK-inhibition is a potent approach to block IgE-mediated**

**histamine release in human basophils**

**Supplementary Tables**

**Supplementary Table S1**

Specification of Antibodies (Ab)

-----------------------------------------------------------------------------------------------------------------------------------------------------------------------------

Antigen	CD	Ab/Clone	Source	Isotype	Conjugate	Provider

**-----------------------------------------------------------------------------------------------------------------------------------------------------------------------------**

Isotype control	n.c.	MOPC-21	mouse	IgG1	PE	BD Biosciences

Isotype control	n.c.	MOPC-21	mouse	IgG1	FITC	BD Biosciences

aminopeptidase N	CD13	WM15	mouse	IgG1	PE	BD Biosciences

LAMP-3	CD63	CLB-gran12	mouse	IgG1	FITC	Immunotech

Sialomucin core protein 24	CD164	67D2	mouse	IgG1	PE	BioLegend

E-NPP3	CD203c	97A6	mouse	IgG1	PE	Immunotech

E-NPP3	CD203c	FR3-16A11	mouse	IgG1	APC	Miltenyi

Annexin V	n.c.	n.a.	n.a.	n.a.	FITC	eBiosciences

Active Caspase-3	n.c.	C92-605	mouse	IgG1	PE	BD Biosciences

pAKT	n.c.	M89-61	mouse	IgG1	PE	BD Biosciences

pBTK	n.c.	N35-86	mouse	IgG1	AlexaFluor-647	BD Biosciences

pS6	n.c.	N7-548	mouse	IgG1	AlexaFluor-647	BD Biosciences

pSTAT5	n.c.	47	mouse	IgG1	AlexaFluor-647	BD Biosciences

pSYK	n.c.	I120-722	mouse	IgG1	PE	BD Biosciences

**------------------------------------------------------------------------------------------------------------------------------------------------------------------------------**

CD, cluster of differantiation; n.a., not available; n.c., not clustered; LAMP-3, Lysosome-associated membrane glycoprotein 3; E-NPP3, Ectonucleotide Pyrophosphatase/Phosphodiesterase 3; pAKT, phosphorylated protein kinase B (PKB), also known as Akt; pBTK, phosphorylated Bruton's tyrosine kinase; pS6, phosphorylated ribosomal protein S6; pSTAT5, phosphorylated signal transducer and activator of transcription 5; pSYK, phosphorylated spleen tyrosine kinase; IgG, immunoglobulin G; PE, phycoerythrin; FITC, fluorescein isothiocyanate; APC, allophycocyanin; Location of providers: BD Biosciences, San José, CA, USA; R&D Systems, Minneapolis, MN, USA; Immunotech, Marseille, France; BioLegend, San Diego, CA, USA; Miltenyi Biotec GmbH, Bergisch Gladbach, Germany; eBiosciences, San Diego, CA, USA.

**Supplementary Table S2**

Target kinase interaction profiles of pharmacologic inhibitors used in this study

-----------------------------------------------------------------------------------------------------------------------------------------------------------------

Drug	Supplier	Major known kinase targets*

-----------------------------------------------------------------------------------------------------------------------------------------------------------------

Dasatinib	ChemieTek	**BTK**, PDGFRs, ABL, SRC, KIT, FYN, TEC, LYN

Ibrutinib	Selleck	**BTK**, BLK, YES, ITK, TEC, ABL, FYN, LYN

AVL-292	Selleck	**BTK**, JAK3, BLK, TEC, ITK

CNX-774	Selleck	**BTK** (information concerning other targets is not available)

P505-15	Axon Medchem	SYK, YES, LYN

-----------------------------------------------------------------------------------------------------------------------------------------------------------------

*Interacting targeted kinases reported in the available literature.

Abbreviations: BTK, Bruton’s tyrosine kinase; PDGFRs, platelet-derived growth factor receptors; ABL, Abelson murine leukemia viral oncogene homolog; SRC, sarcoma; KIT, tyrosine-protein kinase Kit/stem cell growth factor receptor; TEC, Tyrosine-protein kinase Tec; BLK, B lymphocyte kinase; ITK, interleukin-2-inducible T-cell kinase; JAK3, Janus kinase 3; SYK, Spleen tyrosine kinase.

**Supplementary Table S3**

Patients´ characteristics and effects of ibrutinib on allergen-induced histamine release in basophils

-----------------------------------------------------------------------------------------------------------------------------------------------------------------

Diagnosis/Allergy	Age	Allergen-specific IgE levels (ISU)	Ibrutinib IC_50_ (µM)

No.	sensitized against:	f/m	yrs	Phl p 5	Der p 2	Phl p 5	Der p 2

---------------------------------------------------------------------------------------------------------------------------------------------------------------

1	Birch/Timothy grass pollen/

House dust mite	m	28	5.10	6.32	0.023	n.t.

2	Birch pollen/House dust mite	f	24	0.0	27.59	n.t.	0.007

3	Timothy grass pollen/House dust mite	m	23	5.97	2.1	0.005	n.t.

4	Timothy grass pollen	m	38	19.39	0.0	0.015	n.t.

5	Timothy grass pollen	f	56	4.0	0.0	n.t.	n.t.

6	House dust mite	f	27	0.0	78.98	n.t.	n.t.

7	House dust mite	f	23	0.0	43.63	n.t.	0.003

8	House dust mite	m	31	0.0	0.69	n.t.	0.008

9.	Timothy grass pollen/

House dust mite	m	28	54.0	37.16	n.t.	n.t.

10.House dust mite	m	29	0.0	15.72	n.t.	n.t.

11.Timothy grass pollen/

House dust mite	w	22	33.49	10.91	n.t.	n.t.

-----------------------------------------------------------------------------------------------------------------------------------------------------------------

Dextran-enriched BA obtained from patients allergic to Phl p 5 and/or Der p 2 were pre-incubated in various concentrations of ibrutinib (0.001-1 µM) for 30 minutes at 37°C. Then, drug-exposed BA were incubated with recombinant Der p 2 and/or Phl p 5 (each 1 µg/ml) or control-buffer (HRB) for 30 minutes. After incubation, cells were centrifuged at 4°C, and the cell-free supernatants and total suspensions recovered and analyzed for histamine content by radioimmunoassay. Histamine release was calculated as percent of total histamine. All experiments were performed in triplicates. Values represent the half-maximal inhibitory concentration (IC_50_) of ibrutinib.

Abbreviations: f, female; m, male; ISU, ISAC standardized units (for IgE-detection); ISAC, immuno-solid phase allergen chip; n.t., not tested. ISU were determined with the ´mechanisms for the development of allergies (MeDALL) allergen-chip´; Phl p 5, timothy grass pollen; Der p 2, house dust mite. IC50 values were determined using histamine release assay. MeDALL allergen-chip scoring system: <0.3 ISU undetectable or very low, ≥0.3 - <1 ISU low, ≥1 - ≤10 ISU moderate to high, >10 ISU very high.

**Supplementary Table S4**

Non-allergic donors and basophil responses to BTK inhibitors in histamine release experiments

--------------------------------------------------------------------------------------------------------

Age	Ibrutinib	AVL-292	CNX-774

No.	f/m	yrs	IC_50_ (µM)	IC_50_ (µM)	IC_50_ (µM)

--------------------------------------------------------------------------------------------------------

1	m	50	0.005	0.037	0.080

2	f	37	0.03	0.092	0.247

3	f	33	0.003	0.038	0.195

4	m	26	n.t	n.t	n.t

5	f	38	n.t.	n.t	n.t

--------------------------------------------------------------------------------------------------------

Dextran-enriched BA were incubated in the presence or absence of various concentrations of targeted drugs (ibrutinib, AVL-292, CNX-774) (0.001-1 µM) for 30 minutes at 37°C, and thereafter incubated with anti-IgE antibody E124.2.8 (1 µg/ml) in histamine release buffer (HRB) at 37°C for another 30 minutes. After incubation, cells were centrifuged at 4°C, and the cell-free supernatants and total suspensions recovered and analyzed for histamine content by radioimmunoassay. Histamine release was calculated and expressed as percent of total histamine. All experiments were performed in triplicates. Values represent the half-maximal inhibitory concentration (IC_50_) of the tested drugs.

Abbreviations: f, female; m, male; IC_50_, half-maximal inhibitory concentrations; n.t., not tested.

**Supplementary Table S5**

Patients´ characteristics and effects of BTK inhibitors on allergen-induced histamine release in basophils

-------------------------------------------------------------------------------------------------------------------------------------------------------------

Diagnosis/Allergy	Ibrutinib IC_50_ (µM)	AVL-292 IC_50_ (µM)	CNX-774 IC_50_ (µM)	Dasatinib IC_50_ (µM)

No.	sensitized against:	Phl p 5	Der p 2	Phl p 5	Der p 2	Phl p 5	Der p 2	Phl p 5	Der p 2

-------------------------------------------------------------------------------------------------------------------------------------------------------------

1	Birch/Timothy grass pollen/

House dust mite	0.023	n.t.	n.t.	n.t.	n.t.	n.t.	n.t.	n.t.

2	Birch pollen/House dust mite	n.t.	0.007	n.t.	0.073	n.t.	0.298	n.t.	n.t.

3	Timothy grass pollen/

House dust mite	0.005	n.t.	0.091	n.t	0.048	n.t.	0.310	n.t.

4	Timothy grass pollen	0.015	n.t.	0.141	n.t.	0.270	n.t.	n.t.	n.t.

5	Timothy grass pollen	n.t.	n.t.	0.0008	n.t.	0.0007	n.t.	n.t.	n.t.

6	House dust mite	n.t.	n.t.	n.t.	0.056	n.t.	0.048	n.t.	n.t.

7	House dust mite	n.t.	0.003	n.t.	n.t.	n.t.	n.t	n.t.	n.t.

8	House dust mite	n.t.	0.008	n.t.	0.030	n.t.	0.240	n.t.	0.180

9.	Timothy grass pollen/

House dust mite	n.t.	n.t.	n.t.	n.t.	n.t.	n.t.	0.192	0.513

10.House dust mite	n.t.	n.t.	n.t.	n.t.	n.t.	n.t.	0.187	n.t.

11.Timothy grass pollen/

House dust mite	n.t.	n.t.	n.t.	n.t	n.t.	n.t.	0.044	n.t.

-------------------------------------------------------------------------------------------------------------------------------------------------------------

Dextran-enriched BA obtained from patients allergic to Phl p 5 and/or Der p 2 were pre-incubated in various concentrations of ibrutinib, AVL-292 and/or CNX-774 (0.001-1 µM) and/or dasatinib (0.001-1 µM) for 30 minutes at 37°C. Then, drug-exposed BA were incubated with recombinant Der p 2 and/or Phl p 5 (each 1 µg/ml) or control-buffer for 30 minutes. After incubation, cells were centrifuged at 4°C, and the cell-free supernatants and total suspensions recovered and analyzed for histamine content by RIA. Histamine release was calculated as percent of total histamine. All experiments were performed in triplicates. Values represent IC_50_ values produced by ibrutinib, AVL-292, CNX-774, or dasatinib. Abbreviations: f, female; m, male; ISU, ISAC standardized units (for IgE-detection); ISAC, immuno-solid phase allergen chip; n.t., not tested. ISU were determined with the ´mechanisms for the development of allergies (MeDALL) allergen-chip´; Phl p 5, timothy grass pollen; Der p 2, house dust mite. IC_50_ values were determined using histamine release assay. MeDALL allergen-chip scoring system: <0.3 ISU undetectable or very low, ≥0.3 - <1 ISU low, ≥1 - ≤10 ISU moderate to high, >10 ISU very high.

**Supplementary Figures**

**Supplementary Figure S1A**

**Supplementary Figure S1B**

**Supplementary Figure S1C**

**Supplementary Figure S1D**

**Supplementary Figure S1: Effects of ibrutinib on expression of pSyk, pS6, pSTAT5 and pAkt in HMC-1 cells and KU812 cells**

A-D: HMC-1.1 cells (upper left panel), HMC-1.2 cells (upper right panel), and KU812 cells (lower panel) were incubated with control medium (Co) or medium containing ibrutinib, AVL-292, CNX-774, dasatinib or P505-15 (0.1-10 µM) at 37°C for 4 hours. Thereafter, cells were permeabilized and stained with antibodies against pSyk (Y348) (A), pS6 (S235/S236) (B), pSTAT5 (Y694) (C), and pAkt (S473) (D) as described in the text. Expression of phosphorylated (p) signaling molecules in HMC-1 cells, and KU812 cells were determined by flow cytometry. Results show the mean fluorescence intensity expressed as percentage of control and represent the mean±S.D. from 3 independent experiments. Asterisk (*) p<0.05.

**Supplementary Figure S2A**

**Supplementary Figure S2B**

**Supplementary Figure S2: Effects of CNX-774 on survival of HMC-1 and KU812 cells**

A: HMC-1.1 cells (upper left panel), HMC-1.2 cells (lower left panel), and KU812 cells (right panel) were cultured in control medium (Co), or in various concentrations of CNX-774 (0.05-5 µM) at 37°C for 48 hours. Then, cell cycle distribution was analyzed by flow cytometry. Results show relative cell numbers and represent the mean±S.D. from 3 independent experiments. B: HMC-1.1 cells (grey bars), HMC-1.2 cells (open bars) and KU812 cells (black bars) were incubated in control medium (Co) or in medium containing CNX-774 (0.01-10 µM) at 37°C for 48 hours. After incubation, cells were examined for combined AnnexinV/PI staining (left panel) or the percentage of active caspase 3-positive cells was determined (right panel) by flow cytometry. Results represent the mean±S.D. of three independent experiments. Asterisk (*): p<0.05.
