# Supplementary material for: BTK inhibition is a potent approach to block IgE‐mediated histamine release in human basophils
Source: Allergy. 2017 Apr 20;72(11):1666–76. doi: 10.1111/all.13166 (PMC5655929; doi:10.1111/all.13166)
Supplement: Supplementary file 1 [file ALL-72-1666-s001.zip › all13166-sup-0008-TableS1.doc]

**Supplementary Material to Manuscript: Smiljkovic et al:**

**BTK-inhibition is a potent approach to block IgE-mediated**

**histamine release in human basophils**

**Supplementary Table S1**

Specification of Antibodies (Ab)

-----------------------------------------------------------------------------------------------------------------------------------------------------------------------------

Antigen	CD	Ab/Clone	Source	Isotype	Conjugate	Provider

**-----------------------------------------------------------------------------------------------------------------------------------------------------------------------------**

Isotype control	n.c.	MOPC-21	mouse	IgG1	PE	BD Biosciences

Isotype control	n.c.	MOPC-21	mouse	IgG1	FITC	BD Biosciences

aminopeptidase N	CD13	WM15	mouse	IgG1	PE	BD Biosciences

LAMP-3	CD63	CLB-gran12	mouse	IgG1	FITC	Immunotech

Sialomucin core protein 24	CD164	67D2	mouse	IgG1	PE	BioLegend

E-NPP3	CD203c	97A6	mouse	IgG1	PE	Immunotech

E-NPP3	CD203c	FR3-16A11	mouse	IgG1	APC	Miltenyi

Annexin V	n.c.	n.a.	n.a.	n.a.	FITC	eBiosciences

Active Caspase-3	n.c.	C92-605	mouse	IgG1	PE	BD Biosciences

pAKT	n.c.	M89-61	mouse	IgG1	PE	BD Biosciences

pBTK	n.c.	N35-86	mouse	IgG1	AlexaFluor-647	BD Biosciences

pS6	n.c.	N7-548	mouse	IgG1	AlexaFluor-647	BD Biosciences

pSTAT5	n.c.	47	mouse	IgG1	AlexaFluor-647	BD Biosciences

pSYK	n.c.	I120-722	mouse	IgG1	PE	BD Biosciences

**------------------------------------------------------------------------------------------------------------------------------------------------------------------------------**

CD, cluster of differantiation; n.a., not available; n.c., not clustered; LAMP-3, Lysosome-associated membrane glycoprotein 3; E-NPP3, Ectonucleotide Pyrophosphatase/Phosphodiesterase 3; pAKT, phosphorylated protein kinase B (PKB), also known as Akt; pBTK, phosphorylated Bruton's tyrosine kinase; pS6, phosphorylated ribosomal protein S6; pSTAT5, phosphorylated signal transducer and activator of transcription 5; pSYK, phosphorylated spleen tyrosine kinase; IgG, immunoglobulin G; PE, phycoerythrin; FITC, fluorescein isothiocyanate; APC, allophycocyanin; Location of providers: BD Biosciences, San José, CA, USA; R&D Systems, Minneapolis, MN, USA; Immunotech, Marseille, France; BioLegend, San Diego, CA, USA; Miltenyi Biotec GmbH, Bergisch Gladbach, Germany; eBiosciences, San Diego, CA, USA.
