# Supplementary material for: BTK inhibition is a potent approach to block IgE‐mediated histamine release in human basophils
Source: Allergy. 2017 Apr 20;72(11):1666–76. doi: 10.1111/all.13166 (PMC5655929; doi:10.1111/all.13166)
Supplement: Supplementary file 1 [file ALL-72-1666-s001.zip › all13166-sup-0009-TableS2.doc]

**Supplementary Material to Manuscript: Smiljkovic et al:**

**BTK-inhibition is a potent approach to block IgE-mediated**

**histamine release in human basophils**

**Supplementary Table S2**

Target kinase interaction profiles of pharmacologic inhibitors used in this study

-----------------------------------------------------------------------------------------------------------------------------------------------------------------

Drug	Supplier	Major known kinase targets*

-----------------------------------------------------------------------------------------------------------------------------------------------------------------

Dasatinib	ChemieTek	**BTK**, PDGFRs, ABL, SRC, KIT, FYN, TEC, LYN

Ibrutinib	Selleck	**BTK**, BLK, YES, ITK, TEC, ABL, FYN, LYN

AVL-292	Selleck	**BTK**, JAK3, BLK, TEC, ITK

CNX-774	Selleck	**BTK** (information concerning other targets is not available)

P505-15	Axon Medchem	SYK, YES, LYN

-----------------------------------------------------------------------------------------------------------------------------------------------------------------

*Interacting targeted kinases reported in the available literature.

Abbreviations: BTK, Bruton’s tyrosine kinase; PDGFRs, platelet-derived growth factor receptors; ABL, Abelson murine leukemia viral oncogene homolog; SRC, sarcoma; KIT, tyrosine-protein kinase Kit/stem cell growth factor receptor; TEC, Tyrosine-protein kinase Tec; BLK, B lymphocyte kinase; ITK, interleukin-2-inducible T-cell kinase; JAK3, Janus kinase 3; SYK, Spleen tyrosine kinase.
