# Supplementary material for: BTK inhibition is a potent approach to block IgE‐mediated histamine release in human basophils
Source: Allergy. 2017 Apr 20;72(11):1666–76. doi: 10.1111/all.13166 (PMC5655929; doi:10.1111/all.13166)
Supplement: Supplementary file 1 [file ALL-72-1666-s001.zip › all13166-sup-0010-TableS3.doc]

**Supplementary Material to Manuscript: Smiljkovic et al:**

**BTK-inhibition is a potent approach to block IgE-mediated**

**histamine release in human basophils**

**Supplementary Table S3**

Patients´ characteristics and effects of ibrutinib on allergen-induced histamine release in basophils

-----------------------------------------------------------------------------------------------------------------------------------------------------------------

Diagnosis/Allergy	Age	Allergen-specific IgE levels (ISU)	Ibrutinib IC_50_ (µM)

No.	sensitized against:	f/m	yrs	Phl p 5	Der p 2	Phl p 5	Der p 2

-----------------------------------------------------------------------------------------------------------------------------------------------------------------

1	Birch/Timothy grass pollen/

House dust mite	m	28	5.10	6.32	0.023	n.t.

2	Birch pollen/House dust mite	f	24	0.0	27.59	n.t.	0.007

3	Timothy grass pollen/House dust mite	m	23	5.97	2.1	0.005	n.t.

4	Timothy grass pollen	m	38	19.39	0.0	0.015	n.t.

5	Timothy grass pollen	f	56	4.0	0.0	n.t.	n.t.

6	House dust mite	f	27	0.0	78.98	n.t.	n.t.

7	House dust mite	f	23	0.0	43.63	n.t.	0.003

8	House dust mite	m	31	0.0	0.69	n.t.	0.008

9.	Timothy grass pollen/

House dust mite	m	28	54.0	37.16	n.t.	n.t.

10.House dust mite	m	29	0.0	15.72	n.t.	n.t.

11.Timothy grass pollen/

House dust mite	w	22	33.49	10.91	n.t.	n.t.

-----------------------------------------------------------------------------------------------------------------------------------------------------------------

Dextran-enriched BA obtained from patients allergic to Phl p 5 and/or Der p 2 were pre-incubated in various concentrations of ibrutinib (0.001-1 µM) for 30 minutes at 37°C. Then, drug-exposed BA were incubated with recombinant Der p 2 and/or Phl p 5 (each 1 µg/ml) or control-buffer (HRB) for 30 minutes. After incubation, cells were centrifuged at 4°C, and the cell-free supernatants and total suspensions recovered and analyzed for histamine content by radioimmunoassay. Histamine release was calculated as percent of total histamine. All experiments were performed in triplicates. Values represent the half-maximal inhibitory concentration (IC_50_) of ibrutinib.

Abbreviations: f, female; m, male; ISU, ISAC standardized units (for IgE-detection); ISAC, immuno-solid phase allergen chip; n.t., not tested. ISU were determined with the ´mechanisms for the development of allergies (MeDALL) allergen-chip´; Phl p 5, timothy grass pollen; Der p 2, house dust mite. IC50 values were determined using histamine release assay. MeDALL allergen-chip scoring system: <0.3 ISU undetectable or very low, ≥0.3 - <1 ISU low, ≥1 - ≤10 ISU moderate to high, >10 ISU very high.
