# Supplementary material for: BTK inhibition is a potent approach to block IgE‐mediated histamine release in human basophils
Source: Allergy. 2017 Apr 20;72(11):1666–76. doi: 10.1111/all.13166 (PMC5655929; doi:10.1111/all.13166)
Supplement: Supplementary file 1 [file ALL-72-1666-s001.zip › all13166-sup-0011-TableS4.doc]

**Supplementary Material to Manuscript: Smiljkovic et al:**

**BTK-inhibition is a potent approach to block IgE-mediated**

**histamine release in human basophils**

**Supplementary Table S4**

Non-allergic donors and basophil responses to BTK inhibitors in histamine release experiments

--------------------------------------------------------------------------------------------------------

Age	Ibrutinib	AVL-292	CNX-774

No.	f/m	yrs	IC_50_ (µM)	IC_50_ (µM)	IC_50_ (µM)

--------------------------------------------------------------------------------------------------------

1	m	50	0.005	0.037	0.080

2	f	37	0.03	0.092	0.247

3	f	33	0.003	0.038	0.195

4	m	26	n.t	n.t	n.t

5	f	38	n.t.	n.t	n.t

--------------------------------------------------------------------------------------------------------

Dextran-enriched BA were incubated in the presence or absence of various concentrations of targeted drugs (ibrutinib, AVL-292, CNX-774) (0.001-1 µM) for 30 minutes at 37°C, and thereafter incubated with anti-IgE antibody E124.2.8 (1 µg/ml) in histamine release buffer (HRB) at 37°C for another 30 minutes. After incubation, cells were centrifuged at 4°C, and the cell-free supernatants and total suspensions recovered and analyzed for histamine content by radioimmunoassay. Histamine release was calculated and expressed as percent of total histamine. All experiments were performed in triplicates. Values represent the half-maximal inhibitory concentration (IC_50_) of the tested drugs.

Abbreviations: f, female; m, male; IC_50_, half-maximal inhibitory concentrations; n.t., not tested.
